# A Deficit in Movement-Derived Sentences in German-Speaking Hearing-Impaired Children

**DOI:** 10.3389/fpsyg.2017.00689

**Published:** 2017-06-13

**Authors:** Esther Ruigendijk, Naama Friedmann

**Affiliations:** ^1^Department of Dutch and Cluster of Excellence “Hearing for All”, University of OldenburgOldenburg, Germany; ^2^Language and Brain Lab, Tel Aviv UniversityTel Aviv, Israel

**Keywords:** syntax, hearing impaired children, German, relative clauses, Wh-questions

## Abstract

Children with hearing impairment (HI) show disorders in syntax and morphology. The question is whether and how these disorders are connected to problems in the auditory domain. The aim of this paper is to examine whether moderate to severe hearing loss at a young age affects the ability of German-speaking orally trained children to understand and produce sentences. We focused on sentence structures that are derived by syntactic movement, which have been identified as a sensitive marker for syntactic impairment in other languages and in other populations with syntactic impairment. Therefore, our study tested subject and object relatives, subject and object Wh-questions, passive sentences, and topicalized sentences, as well as sentences with verb movement to second sentential position. We tested 19 HI children aged 9;5–13;6 and compared their performance with hearing children using comprehension tasks of sentence-picture matching and sentence repetition tasks. For the comprehension tasks, we included HI children who passed an auditory discrimination task; for the sentence repetition tasks, we selected children who passed a screening task of simple sentence repetition without lip-reading; this made sure that they could perceive the words in the tests, so that we could test their grammatical abilities. The results clearly showed that most of the participants with HI had considerable difficulties in the comprehension and repetition of sentences with syntactic movement: they had significant difficulties understanding object relatives, Wh-questions, and topicalized sentences, and in the repetition of object *who* and *which* questions and subject relatives, as well as in sentences with verb movement to second sentential position. Repetition of passives was only problematic for some children. Object relatives were still difficult at this age for both HI and hearing children. An additional important outcome of the study is that not all sentence structures are impaired—passive structures were not problematic for most of the HI children

## Introduction

Children with hearing impairment (HI) very often show language problems. Many studies of the language of HI children examine their vocabulary and phonology, and demonstrate difficulties in these language domains (e.g., Davis et al., 1986; Briscoe et al., 2001). In the current study, we focus on a different language domain of great difficulty in HI children: syntax. The ability to understand and produce sentences is a core language ability, but studies have shown that children with HI show great difficulty in syntax, in both the comprehension and production of syntactically complex sentences (Pressnell, 1973; Sarachan-Deily and Love, 1974; Geers and Moog, 1978; Berent, 1996; Brannon, 1966, 1968; Quigley and King, 1980; Friedmann and Szterman, 2006, 2011; Delage and Tuller, 2007; Geers et al., 2009; Friedmann and Costa, 2011; Friedmann and Haddad-Hanna, 2014; Szterman and Friedmann, 2014b).

Studies that examined which sentence structures pose difficulties to HI children, done mainly in English, Hebrew, and Arabic, pointed to several structures that are especially difficult for these children. These were mainly Wh-questions, object relatives, object topicalization sentences, and passive sentences.

**Wh-questions**, like “which girl did grandma draw?” were found to be impaired in HI children’s comprehension and production (Quigley et al., 1974b; Geers and Moog, 1978; de Villiers, 1988; de Villiers et al., 1994; Berent, 1996; Friedmann et al., 2010b; Friedmann and Szterman, 2011; Szterman and Friedmann, 2015). **Relative clauses**, such as “this is the girl who grandma kissed” were also found to cause special difficulty for HI children in both comprehension and production (Quigley et al., 1974a; Berent, 1988; de Villiers, 1988; Friedmann and Szterman, 2006; Friedmann et al., 2008, 2010b; Friedmann and Haddad-Hanna, 2014; Szterman and Friedmann, 2014a, 2015; Volpato and Vernice, 2014). Similar difficulties have also been reported for **topicalization** structures, such as “this girl, the grandma loved” (Friedmann and Szterman, 2006; Szterman and Friedmann, 2014a, 2015). A further type of sentences that was reported to be difficult for HI children is the passive construction, such as “the girl was tickled by the grandma” (Power and Quigley, 1973).

Syntactically, these structures share a common property—they are all derived by syntactic movement. Syntactic movement is the operation that creates a structure by movement of an element from a basic word order (also termed the base-generated order). For instance, it is assumed that in English (and other languages) the basic word order is subject-verb-object. To derive the topicalized structure “this girl, the grandma loved” from the base-generated order “the grandma loved this girl”, *this girl* is moved from a position after the verb *loved* to the first position of the sentence. It has therefore been argued that HI children may have a specific problem with structures that are derived by syntactic movement (see e.g., Friedmann and Szterman, 2006, 2011).

Within the movement-derived sentence structures, the structures in which HI children show most difficulties are the ones where the order of the participants in the sentence is not the usual one. In English, Hebrew, and Arabic, where syntax of HI children has been tested, the basic word order (see the simple sentence in 1) is subject-verb-object, or to use the thematic structure: agent-before-theme (note that this is not the same thing, see the discussion on example 10 below). Namely, the agent of the verb (and of the action described in the sentence) precedes the theme of the verb. The movement-derived sentences that are most difficult for HI children to understand, exemplified in 2–5, are the ones where the theme precedes the agent (in 2–5, the grandfather, who is the *theme*, precedes the boy, who is the *agent*).

(1) Simple active sentence: The boy is tickling the grandfather.
(2) Object Wh question: Which grandfather did the boytickle __?
(3) Object relative clause: This is the grandfather that the boy tickles __.
(4) Object topicalization: This grandfather, the boy tickled __.
(5) Passive: The grandfather is tickled __ by the boy.

Sentences (2)–(5) differ in structure, but in all of them *the boy* is the agent of the action (i.e., the tickler), and *the grandfather* is the theme of the action (i.e., the one being tickled). The verb *tickle* assigns two thematic roles: the role of *agent* to the noun phrase (NP) that performs the action and *theme* to the NP that receives the action or is affected by it. The assignment of these thematic roles is done according to the base-generated order: the verb assigns the agent role to the NP that precedes it and the theme role to the verb that follows it. Since in sentences (2–5) the object is moved to the position before the verb, the question is how this NP receives its thematic role. Within Government and Binding theory (Chomsky, 1981) it is assumed that NPs that move, leave behind a trace in their original position (marked by an underlined gap in examples 2–5). The verb assigns the thematic role to the trace of the moved NP and the role is then transferred from the trace position to the moved constituent through a chain consisting of the trace and the moved NP. For (2–5) this means that the verb assigns a thematic role of *theme* to the trace of the NP *the grandfather*, which has moved. This role is then transferred to *the grandfather*, through a chain of movement, and hence this NP can be interpreted as the theme of the sentence. In processing terms, one may think of movement as re-activation of the NP that moved in its base-generated position: upon hearing the sentence in (2), for example, the hearer keeps the NP “which grandfather” in a syntactic memory component until she hears the verb, and then she can re-access this NP after the verb, and interpret it as the theme, in order to understand ‘who did what to whom’ in the sentence.

Sentences in which the theme (the object of the sentence here) moves across the agent (the subject) to a position in the beginning of the sentence are especially difficult for various populations: young children who have not yet completed the acquisition of syntax in their language (Friedmann et al., 2009, 2010a; Belletti et al., 2012; Biran and Ruigendijk, 2015), children with developmental syntactic impairment, SySLI (Friedmann and Novogrodsky, 2004, 2011; Friedmann et al., 2015), and individuals with agrammatism (Grodzinsky et al., 1999). In studies of English, Hebrew, and Palestinian Arabic, the difficulty in these structures is cast in terms of word order: the theme moves to a position before the agent, and the word order is not the canonical one; to distinguish between an object and a subject question in English, for example (Which grandfather did the boy tickle vs. Which grandfather tickles the boy), one needs to rely on word order.

The situation is different in German. German marks subjects and objects through morphology, using case-marking. Subject and object-first sentences have the same order of NPs and verbs and only differ in the case-marking of these NPs. German NPs are marked for case, as can be seen in sentence (6), where *der Junge* ‘the boy’ has nominative case and *den Opa* ‘the grandfather’ accusative case.^1^ Sentence (7–9) show German examples of three of the structures with Wh-movement, which have been found to be impaired in children with HI: object Wh-questions, object relatives and topicalized sentences (parallel to the English examples in 2–4).^2^

(6) Simple active:

                               Der Junge kitzelt den Opa.

                               the_NOM_^3^ boy tickles the_ACC_ grandfather.

                               ‘The boy is tickling the grandfather.’

(7) Object Wh-question:

                               Welchen Opa_1_ kitzelt der Junge *t*_1_?

                               which_ACC_ grandfather tickles the_NOM_ boy?

                               ‘Which grandfather does the boy tickle?’

(8) Object relative clause:

                               Das ist der Opa_1_, den der Junge *t*_1_ kitzelt.

                               this is the_NOM_ grandfather, that_ACC_ the_NOM_ boy tickles.

                               ‘This is the grandfather that the boy tickles’

(9) Topicalization:

                               Den Opa_1_ kitzelt der Junge *t*_1_.

                               the_ACC_ grandfather tickles the_NOM_ boy.

                               ‘It is the grandfather that the boy tickles.’

                               (The German sentence does not include embedding, but this translation keeps the gist of the use of such sentences.)

In German, case morphology gives important information as to ‘who did what to whom’. In our sentences (6–9), the subject of the sentence always has nominative case *der Junge* ‘the_NOM_ boy’, marked here on the article of the NP. For masculine NPs, the article always unambiguously distinguishes nominative (der) and accusative (den) case. This marks the subject and object, and hence provides clear information on who does what to whom. Studies on language acquisition in young German-speaking children (up until the age of 7 at least) show that, although object-first sentences are still not comprehended adult-like, such unambiguous case-marking does indeed improve comprehension (Arosio et al., 2012; Biran and Ruigendijk, 2015; Roesch and Chondrogianni, 2015) as well as sentence repetition (Biran and Ruigendijk, 2015).

Thus, in German, correct interpretation and use of these specific structures depends on morphosyntactic information^4^ that is perceptually not very salient: determiners and verbal inflection. However, it does not seem to be the perceptual salience of the case-bearing words that is the source of the difficulty with these syntactic structures in HI. We can see that difficulty in movement-derived sentences is apparent also in languages such as English, Italian, Hebrew and Arabic, where these syntactic structures are not marked by similarly-sounding case markers but rather by (perceptually salient) word order. In addition to morphosyntactic information, the different structures are realized with different prosody. However, difficulties in perceiving prosody cannot be the source of the difficulty either. First of all, people understand sentences with movement even when they are written, when no prosody is provided. Additionally, HI children show similar deficits in written movement-derived sentences (Quigley et al., 1974a; Szterman and Friedmann, 2014a,b), where no prosodic information is provided. This confirms the idea that prosody is not the only aspect that can distinguish these types of sentences, and that there is a special role for morphosyntactic information worth studying in HI children.

It has been shown by Hennies et al. (2012), that German-speaking HI children perform worse than normal-hearing children on the perception of consonants that are relevant for subject-verb agreement on syllable offset. Furthermore, Szagun (2004) showed that the article system of German-speaking children with a CI (cochlear implant) is less well-developed than that in normal hearing children, which she argues is the result of persisting perceptual problems. Steinbrink (2004), however, found for –*n* and vowels (which are important for case morphology – *n* for the distinction between the case-marked determiners *den* and *der* and *dem*; vowels for the distinction between the determiners *die, das*, and *der* vs. *den/dem*) no clear correlation between phonological problems and the production of correct inflectional morphology (as examined through spontaneous speech analysis). Similarly, in one of our own recent (eyetracking) studies, we found that CI children are aware of both case and subject–verb agreement morphology, but show a considerable delay in the effect of this morphosyntactic information on sentence interpretation (Schouwenaars et al., 2015). It is thus especially interesting to examine how German-speaking children with HI understand and produce structures with Wh-movement in which the theme precedes the agent, and which require the processing of case markers, and this is one of the aims of our study.

We have so far discussed ***Wh-movement***, a movement of a phrase to the beginning of the sentence (NP or PP to spec-CP, i.e., the specifier of the complementizer phrase, in syntactic terms), which derives Wh-questions, relative clauses, and topicalization sentences (sentences 7–9). However, Wh-movement is only one type of movement that results in a non-canonical structure. Types of syntactic movement differ by the type of element that moves, and the position to which it moves. Assessing comprehension and repetition of HI in German allows us to explore another question: are all types of movement impaired in HI? We therefore assessed two additional types of movement-derived sentences, in addition to Wh-movement: one is a type of movement that involves the movement of an NP, but to a different sentential position – a movement from object to subject position (which is called *A-movement*), which occurs, for example, in passive sentences such as (10); the other is the movement of the verb to the second sentential position (*verb movement*, or, in more syntactic terms, V-to-C movement), illustrated in (11).

(10) Passive:

                               Der Opa_1_ wurde von dem Jungen *t*_1_ gekitzelt.

                               the_NOM_ grandfather was by the_DAT_ boy tickled.

                               ‘The grandfather was tickled by the boy.’

(11) Verb movement:

                               Jetzt kitzelt_1_ der Junge den Opa *t*_1_.

                               now tickles the_NOM_ boy the_ACC_ grandfather.

                               ‘Now the boy is tickling the grandfather’

In (10), similar to (7–9), the theme, *der Opa*, comes before the agent *der Junge*, that is, the theme has been moved from its original position to the first position of the sentence. Unlike in (7–9), however, it is now the syntactic subject of the sentence, as indicated by subject-verb agreement and as can be seen in its case-marking: nominative. The agent of the sentence is now realized in a ‘by phrase’: *von dem Jungen* ‘by the_DAT_ boy_DAT_’, with unambiguous dative case. So, here we have a subject–object word order, but it is still non-canonical in the sense that the first NP is not the agent of the sentence. In this type of movement, the thematic role is assigned to the original position of the object, whereas nominative is assigned to the moved element.

One final type of movement-derived sentences to be tested here is shown in (11). In German, the finite verb of a sentence moves to the second position of the sentence in main clauses, as can be seen in all examples (6–10) already (see footnote 3). Importantly, when a child repeats a simple active sentence in German, with the order subject-verb, one cannot be completely sure what the underlying structure is that results in this output.^5^ When a German sentence starts with an adverb (A), the verb moves to the second position of the sentence, to a position before the subject, creating an AVSO word order (i.e., Adverb – Verb – Subject – Object). With this sentence type, we can be sure about the underlying structure that is realized: the adverb is moved to Spec-CP, whereas the verb is moved to C. A further difference between this structure and the active sentence (in 1) is that both NPs now come after the verb. The order of the NPs is still canonical agent-theme. This type of movement is called V-to-C movement.

The ability to understand and produce sentences with syntactic movement is a crucial language ability. Our aim was to assess whether the lack of sufficient exposure to natural language from birth affects the ability of German-speaking children with HI to understand and produce (non-canonical) sentences that are derived by syntactic movement. We further asked which types of movement are impaired. Unlike other languages in which syntax of HI children was examined so far, like English, Hebrew, or Arabic, German enables us to study the interaction of word order phenomena with morphosyntactic case-marking. Furthermore, German allows testing of sentences that include object movement without other changes in the sentence (topicalization), and allows us to compare various types of syntactic movement: Wh-movement, A-movement as seen in passives, and verb movement (to C). So, for example, English allows examining passives, Wh-questions and relative clauses, but not V-to-C movement of main verbs, or topicalization without other interfering factors, which can be tested in German. Hebrew and Arabic allow the study of V-to-C movement of main verbs and topicalization, as well as relative clauses and Wh-questions, but passives in these languages are rarely used. Thus, examining these structures in German HI may help us better understand the effects of HI on the acquisition of sentences with syntactic movement, by examining another type of movement, beyond phrasal movement, and by examining the effect of case marking on the processing of sentences derived by Wh-movement. Furthermore, our data may help to better understand the possible psycholinguistic bases of the syntactic impairment in different populations by systematically studying the effects of HI on language acquisition using similar structures that are studied in these other populations with different etiologies such as syntactic SLI or agrammatism.

## General Method

We used two types of tasks to examine the HI children’s syntactic abilities. In the first part of this article we describe two picture selection tasks (Experiments 1 and 2) which we used to test the participants’ comprehension of subject- and object relative clauses and of passive sentences, as well as *who* and *which* subject- and object questions and topicalized sentences. In the second part, we report on two sentence repetition tasks (Experiments 3 and 4) with which we examined subject relative clauses, passive sentences, and subject and object *who* and *which* questions, in comparison with simple SVO sentences (subject–verb–object), and sentences with an adverb (AVSO vs. SVOA). We chose two different types of tasks, comprehension and repetition, to offer converging evidence of a syntactic impairment and to allow for task independent assessment of the difficulty. The picture selection tasks allow for a controlled way of assessing participants’ ability to use syntax for comprehension. Performance on this task is informative in two ways. First, we can test whether the HI children perform similar to or less well than the hearing children. Second, the task allows us to distinguish between above chance, chance and below chance performance, where above-chance performance indicates knowledge of the structure, and chance level or below chance performance suggests that the syntactic information is not acquired yet. Chance performance in the picture selection task would be manifested by random pointing to one of the two pictures, pointing to each picture around half of the time. In our sentence types chance performance suggests that the child is aware of the morphosyntactic information, but cannot yet use it for correct sentence interpretation. Below chance performance means a systematic error pattern, i.e., systematically choosing the distractor picture, which would indicate that the child is not yet aware of the morpho-syntactic information given in the sentence (such as case marking).

Repetition tasks allow full control of the target sentence and the construction of minimal pairs of sentences – one including the tested structure and one completely parallel but without the tested structure. It is hence a relatively simple way to examine the syntactic abilities of children in various structures such as relative clauses, Wh-questions, and passives using the same task. Repeating a sentence in one’s native language involves comprehension and production, and does not merely consist of a passive, phonological copy of the input sentence. Therefore, difficulties in the comprehension and production of a syntactic structure may be manifested in difficulties to repeat this structure (Friedmann and Grodzinsky, 1997; Lust et al., 1998; Potter and Lombardi, 1998; Friedmann, 2006, 2013; Friedmann and Lavi, 2006; Szterman and Friedmann, 2015). When participants repeat sentences that are similar in length and words, which differ only in the relevant syntactic feature tested, and succeed on one structure but fail in the other structure, this might indicate a specific difficulty with the tested structure. Thus, if a child consistently makes structural errors when she repeats a certain structure, but consistently repeats correctly the control sentence, this would indicate that she has not yet mastered this specific structure, or that she has a deficit in this structure. Also, the types of errors that the participant makes when repeating a structure are informative: repetition errors that affect the structure of the sentence indicate a difficulty that is structural in nature. Conversely, lexical errors, i.e., substituting or omitting of the lexical items in a way that does not affect the syntactic structure or the thematic roles in the sentence, may reflect either a lexical difficulty, or the increased difficulty of the target sentence, which might result from its structure. Each task was described in detail below.

Each child was tested individually by a native speaker of German, in 2 to 5 meetings. The children participated at will and they were told that they could stop whenever they wanted. We received informed consent from all parents. No time limit was set in any of the tasks, and the experimenter repeated every item as many times as the participant requested. We varied the type of tasks (i.e., repetition, comprehension^6^) in each session, so that there was enough variation for the child. In between tasks we had short breaks. Apart from that, the child could take as many breaks as s/he wanted. This study was approved by and carried out in accordance with recommendations from the local ethics committee at the University of Oldenburg.

Prior to the experiments, two screening tests were used to assess for each participant (for the HI children: with hearing aid device) whether s/he could perceive language as presented/used in our tests. One screening test was an auditory same-different task, which was designed to make sure that the participants perceived the phonological differences between case inflections, which are crucial for sentence comprehension (and hence also for repetition) in German, and that their performance was not influenced by problems in hearing these morphemes. The participant heard 22 pairs of NPs (each NP including one or two words); The test included pairs of determiners, determiners + nouns, Wh-elements, Wh-element + nouns, and possessive pronoun + N. There were 11 identical pairs and 11 pairs that differed in their case inflection (for instance, identical: *den Jungen – den Jungen* ‘the_MASC,ACC,_ boy_ACC_ - the_MASC,ACC,_ boy_ACC_’; different: *der Esel – den Esel* ‘the_MASC,NOM_ donkey – the_MASC,ACC_ donkey’ _MASC_ = masculine). The participants were asked to judge whether the NPs in each pair were the same or different. Individuals who made errors on more than three items in this screening task did not participate in the study.

The other screening test was a simple sentence repetition task, which was used to make sure that the sentence stimuli in the experiments were perceived correctly, and that the children did not have relevant production difficulties. The experimenter said 10 simple canonical SVO sentences (e.g., *Das junge Mädchen zeichnet den frechen Frosch*. ‘the_NEUT,NOM_ girl draws the_MASC,ACC_ naughty_ACC_ frog’ _NEUT_ = neutral) with her lips concealed, and the participants were asked to repeat each sentence aloud. In this test, omissions and substitutions of the determiners, the nouns, or the verbs were counted as incorrect. We did not count as incorrect errors that resulted from pronunciation difficulties. Individuals who made errors on more than one sentence in this task did not participate in the repetition experiments. Children who did not pass the screening repetition task, but who did well on the same-different task (i.e., less than three errors), did participate in the sentence comprehension tasks, but not in the repetition tasks.

### Participants

In total 24 German-speaking children with HI were examined. Five of them did not pass one or both of the screening tests, and hence their data were not analyzed any further. Four children did pass the same-different task, but not the repetition screening, so they only participated in the sentence comprehension tasks. The children whose data did enter the analysis were nineteen children, 9;5 to 13 years old (*M* = 10;7, *SD* = 0;11), nine girls, ten boys. This age range was chosen (a) since it is important to understand the effects of HI on language performance of school age children, and (b) according to previous studies TD hearing children in this age range acquire most of the syntactic structures by that age.

They had moderate to profound hearing loss, which has been diagnosed at a very young age or relatively late (age range of diagnosis: 0;4–9;0). Fourteen of the children used binaural hearing aids, two used two cochlear implants and three children used one cochlear implant and a hearing aid. Since we were interested in the effect of HI on language impairment in general, we did not distinguish between types of HI. Fourteen of the children went to a special school for children with HI, and the rest attended regular schools. Most of the participants performed all tasks, some of them performed only part of the tasks (see below for details), for organizational reasons. Subject files included no other disabilities, and all children came from a family that spoke only German and that used no sign language. All children were trained orally. All participants constantly wore their hearing aids or their CI(s). The details of each of the participants are presented in Appendix A.

The children in the control groups for these experiments were 96-monolingual typically developing children without language impairment or hearing disorder. They were 7;0–12;5 years old (*M* = 9;9). For organizational reasons, not all hearing children could perform all tasks, see for more details the description of the results below.

### Statistical Analysis

For each task, we ran two types of analyses: group-level and individual-level. The group analysis was done to establish whether HI children in general performed differently from hearing children, that is, whether in general HI causes syntactic difficulties. We were specifically interested in whether in the group some sentence types were more often affected than others. Since it is well-known that there is quite some variation in the performance of the HI children and our group of HI participants was varied in several aspects as well (hearing aids vs CI, age of diagnosis, severity of hearing loss), an individual level analysis was done to further examine the range of abilities and problems in HI children. We were interested in how many and which children performed worse than the hearing group, and whether we could distinguish characteristics in for instance background, or exposure that may explain the difference between good performers and not-so-good performers. We were also interested in whether a scale of difficulty can be detected between the various structures.

We first ran a repeated measures ANOVA with the relevant sentence factors as within subject variable (either: sentence type, or word order and question type), group as between subject-variable and single subject accuracy as dependent variable. For this we used percentage correct so that we could use data from participants for whom we did not have complete data sets^7^. When this resulted in significant effects of group or interactions with group, we ran pairwise comparisons per sentence type to see which sentence type resulted in lower performance in the HI group. This was followed by *post hoc* paired *t*-tests within groups to compare performance on the different sentence types whenever a main effect of sentence type or an interaction of group with sentence type was found. Also for the comprehension tasks, we established whether performance differed from chance or not using the binomial test. Finally, the performance of each individual participant with HI was compared to the control group in each sentence type using the Crawford and Howell’s *t*-test for the comparison of a single participant to a group (Crawford and Howell, 1998; Crawford and Garthwaite, 2002).

## Experiments 1 and 2: Comprehension of Relative Clauses, Passives, Topicalization and Wh-Questions

Sentence comprehension was assessed using two picture selection tasks, one assessed passive sentences and relative clauses compared to simple sentences (Experiment 1); the other (Experiment 2) assessed Wh-questions and topicalization structures in comparison to simple sentences. We used two different tasks to create more variation (and less boredom) for the participants, both regarding method and the pictures we used.

### Material Experiment 1: Comprehension of Relative Clauses and Passives

**FIGURE 1:**
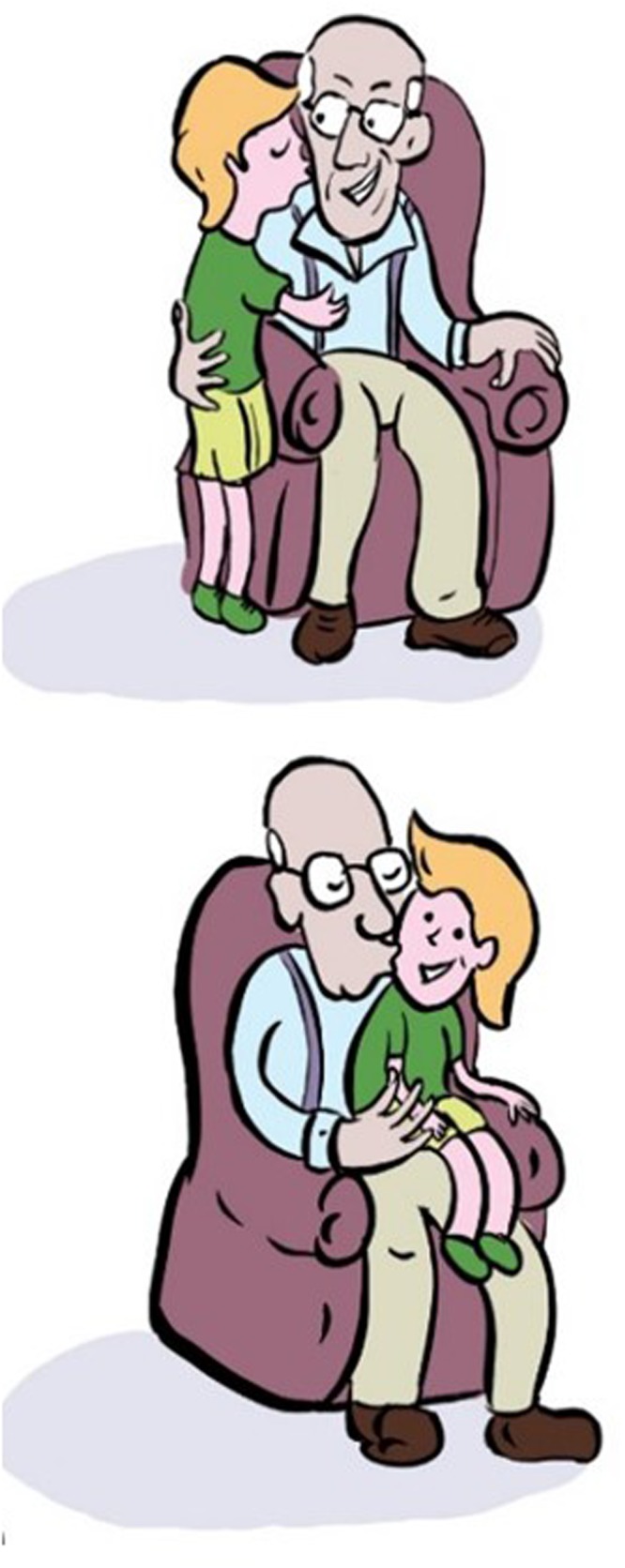
**An example for a picture pair used in Experiment 1**. *Das ist der Junge der den Opa küsst* That is the_MASC,NOM_ boy who_MASC,NOM_ the_MASC,ACC_ grandfather kissed. ‘That’s the boy who kissed the grandfather’

In the first comprehension task, the participant heard a sentence read by a native speaker of German, and saw two pictures on the same page, one above the other. In one picture the roles matched the sentence; in the other picture the roles were reversed (**Figure 1**). The participant was requested to point to the picture that correctly described the sentence.

The task included a total of 80 sentences for each participant, namely 20 simple SVO sentences, 20 subject relatives, 20 object relatives, and 20 passive sentences (see examples in **Table 1**). All verbs were agentive transitive. All the sentences were semantically reversible so that comprehension of the meaning of the words alone cannot determine the meaning of the sentence (namely, we did not use irreversible sentences like ‘*The girl is eating a pear*’, only reversible ones like ‘*The girl is kissing the grandmother’*).

**Table 1:** Types of sentences in Experiment 1.

	Wh movement	Embedding	Example
*Simple SVO*	no	No	Guck mal, der Junge küsst den Opa Look, the_NOM_ boy kisses the_ACC_ grandfather ‘*Look! The boy kisses the grandfather’*
*Passive*	no A-movement	No	Guck mal, der Junge wird vom Opa geküsst Look, the_NOM_ boy is by-the_DAT_ grandfather kissed ‘*Look! The boy is kissed by the grandfather’*
*Subject Relative*	yes agent remains before theme	Yes	Das ist der Junge der den Opa küsst This is the_NOM_ boy that_NOM_ the_ACC_ grandfather kisses ‘*This is the boy that kisses the grandfather’*
*Object Relative*	yes theme moved before agent	Yes	Das ist der Junge den der Opa küsst This is the_NOM_ boy that_ACC_ the_NOM_ grandfather kisses ‘*This is the boy that the grandfather kisses’*

Sentences were randomly ordered, and presented in 2 sessions of 40 sentences each (10 sentences of each type per session). The participants saw the 40 picture pairs twice, once in each session (20 picture pairs were presented with the subject relatives and object relatives, and 20 picture pairs with the SVO and passive sentences, four pictures were used in all four conditions and hence presented twice in each session). The correct picture in each pair was randomized both within a session (in each session half of the sentences matched the upper picture, and half matched the bottom picture), and between sessions (the matching picture in each pair was sometimes the top picture, and sometimes the bottom picture).

For relative clauses, both NPs were masculine, in order to make them unambiguously case-marked (see above). For simple SVO and the passive sentences we used NPs of all three grammatical genders: masculine, feminine, and neutral; 13 of the 20 SVO sentences and 13 of the 20 passive sentences included two NPs of the same gender (in German gender agreement is not marked on the verb).

### Material Experiment 2: Comprehension of Topicalization and Wh-Questions

**FIGURE 2:**
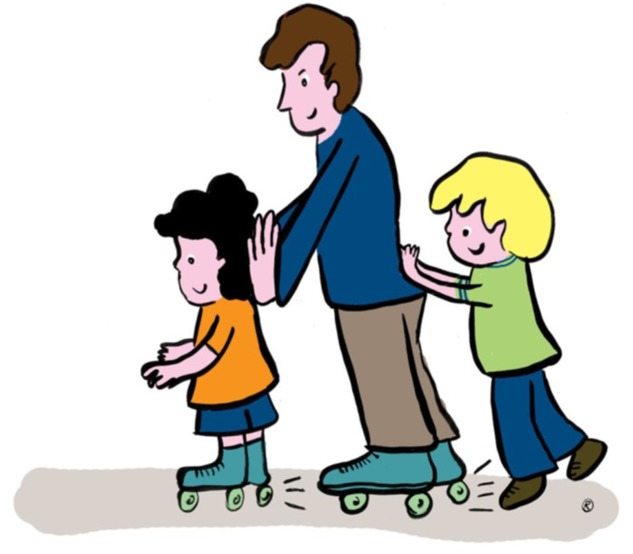
**An example for a picture pair used in Experiment 2**. *Welcher Junge schubst den Mann? Which_NOM,MASC_ boy pushes the_ACC,MASC_ man? ‘Which boy is pushing the man?’*

In the second sentence comprehension task, each sentence was presented with one picture depicting three figures involved in one action (as in **Figure 2**). In the picture, there were two similar figures and one of a different kind (two boys and a man, two elephants and a boy, two clowns and a boy). One of the similar figures was acting upon the figure in the middle, which, in turn, was acting upon the other similar figure. This type of sentence picture matching task is felicitous for examining comprehension of questions (see Hamburger and Crain, 1982 for the importance of felicity in assessing Wh-movement; see Friedmann and Novogrodsky, 2011 for a discussion of the felicity of this specific type of task for assessing comprehension of Wh-questions). For example, in **Figure 2**, a boy in a green shirt is pushing a man who is pushing a boy in an orange shirt. Here too, the experimenter –a native speaker of German- read out a sentence, while the participant saw the picture. The participant then had to point to the correct figure, or alternatively reply orally, by naming the color (e.g., in **Figure 2**: “the green one”, “the boy with the green shirt”).

The test consisted of 108 sentences in 6 conditions, with 18 items in each condition. The sentence types included subject and object *who* and *which* questions and topicalized OVS (object–verb–subject) sentences, as well as simple SVO sentences for comparison (See **Table 2** for examples). Again, sentences were randomly ordered, and presented in 2 sessions of 54 sentences each (9 sentences of each type per session). The participant saw 18 pictures 6 times; three times in each session. The position of the correct actor in each sentence, left or right from the middle figure, was randomized within a session and between sessions.

**Table 2:** Types of sentences in Experiment 2.

	Wh Movement	Embedding	Example
Simple SVO	No	No	Der Junge schubst den Mann
			the_NOM_ boy pushes the_ACC_ man ‘
			*The boy pushes the man.’*
Topicalization OVS	Theme moved before agent	No	Den Mann schubst der Junge
			the_ACC_ man pushes the_NOM_ boy
			‘*It is the man that the boy is pushing’*
Subject who question	Agent remains before theme	No	Wer schubst den Mann?
			Who_NOM_ pushes the_ACC_ man?
			‘who is pushing the man?’
Object *who* question	Theme moved before agent	No	Wen schubst der Junge?
			Who_ACC_ pushes the_NOM_ boy?
			‘Who did the boy push?’
Subject *which* question	Agent remains before theme	No	Welcher Junge schubst den Mann?
			which_NOM_ boy pushes the_ACC_ man?
			‘*Which boy is pushing the man?’*
Object *which* question	Theme moved before agent	No	Welchen Jungen schubst der Mann?
			Which_ACC_ boy_ACC_ pushes the_NOM_ man?
			‘*Which boy is the man pushing?’*

For all sentences, both NPs were masculine. Using feminine or neuter NPs would make the *who* questions structurally ambiguous between subject- and object-question interpretation.

## Results: Comprehension of Relative Clauses, Passives, Topicalization and Wh-Questions

### Experiment 1: Comprehension of Relative Clauses and Passives

**FIGURE 3:**
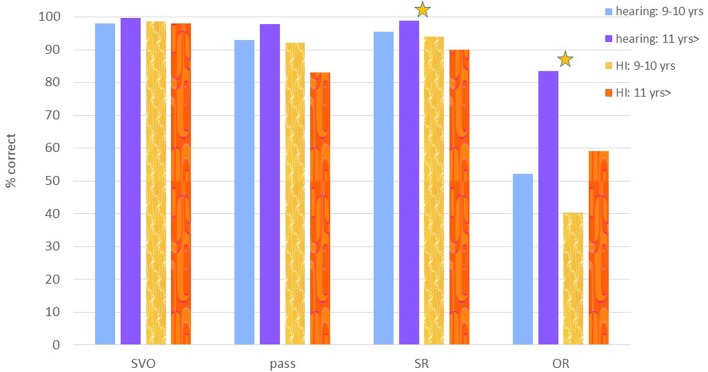
**% correct on Experiment 1: comprehension of SVO (subject–verb–object): simple active sentence; pass: passive sentence; SR and OR: subject and object relative**. A star indicates a significant difference between the (age matched) hearing and HI groups.

The results of Experiment 1 are summarized in **Figure 3**. This task was performed by 19 HI children (age 9;3–13;0, mean 10;7), and by 53 hearing children (age 9;3–12;6, mean 10;8). We analyzed the data with a repeated measure with variables group and sentence type. This revealed a main effect for sentence type [*F*(3,210) = 100.21, *p* < 0.001], caused by overall lower performance on object relatives. We also found a main effect of group [*F*(1,70) = 7.13, *p* = 0.009], and an interaction of group and sentence type [*F*(3,210) = 3.55, *p* = 0.02], caused by lower performance of the hearing impaired group, who performed even worse on the object relatives. *Post hoc* pairwise comparisons (Bonferroni corrected) revealed that SVO sentences overall were comprehended better than each of the three other conditions (*p* < 0.01), and passives and subject relatives were comprehended better than object relatives (*p* < 0.01). A comparison of the performance of the two groups per sentence type (independent *t*-tests) showed that the HI children performed significantly poorer than the hearing control group on subject- and object relatives (*p* = 0.036 and *p* = 0.025, respectively). The hearing children, as a group, performed above chance level on all four conditions (one sample *t*-test *p* < 0.05), whereas the HI children, as a group, performed not differently from chance level on the object relatives (one sample *t*-test, *p* = 0.56), and above chance on the three other conditions.

The hearing group was divided into two age groups: 34 nine and ten year olds (aged 9;3–10;11, including 11 nine year olds and 23 ten year olds), and 19 eleven and twelve year olds (aged 11–12;6).

As shown in **Figure 3**, the comprehension of object relatives in German still develops within the ages we tested: the average performance of hearing children below age 11 was 52% correct, and it improved to 83% in the hearing children who were 11 years old and older. We therefore compared the individual HI participants to the hearing participants by age, comparing the 14 HI participants under the age of 11 to the 9–10 year old hearing children, and the 5 HI children who were older than 11 to the 11–12 year old hearing participants. Comparisons of each of the HI children to her/his age-matched hearing group and to chance level in each sentence type are summarized in **Table 3**. As summarized in **Table 3**, object relative was the structure that showed the most impaired performance in the HI group in this task, with 7 HI participants performing significantly below the matched hearing group, and almost all HI performing not above chance level.

**Table 3:** Number of HI participants performing significantly below the hearing group, and number of HI participants performing not above chance (at/below chance) in the two comprehension experiments.

	Comprehension 1 (no. out of 19 participants)				Comprehension 2 (no. out of 16 participants)					
	SVO	Passive	Subject relative	Objective relative	SVO	OVS	Subject *who*	Object *who*	Subject *which*	Object *which*
No. of HI below hearing group	2	2	3	7	2	6	5	3	6	4
No. of HI below/at chance	0	2	1	15	0	12	0	6	0	9

*Comparison to the control group using Crawford and Howell’s *t*-test, all *p* < 0.05. Comparison to chance level using binom, all *p* < 0.05*.

These results suggest that some of the participants with HI have a considerable difficulty in the comprehension of object relatives, beyond the difficulty their hearing age-peers show. However, the results bear an additional type of important information: that not all types of movement are equally difficult for HI children. Firstly, the passive construction, which involves movement other than Wh-movement, seems to be normally comprehended for most HI children. Secondly, subject relatives, which involve Wh-movement but in which the theme does not cross the agent in its movement, is also comprehended relatively well. These findings thus suggest a selective deficit affecting object Wh-dependencies in children with HI.

### Experiment 2: Comprehension of Topicalization and Wh-Questions

**FIGURE 4:**
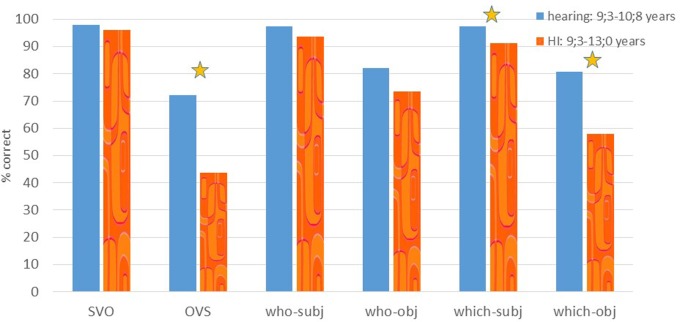
**% correct on Experiment 2: comprehension of SVO, topicalization, subject and object *who* and *which* questions**. A star indicates a significant difference between the groups.

The results of Experiment 2 are summarized in **Figure 4**. This task was performed by 16 HI children (aged 9;3–13;0, mean 10;6), and 18 hearing children whose ages were similar to the youngest children in the HI group (aged 9;3–10;8, mean 9;10).

We ran two separate repeated measure analyses, one to compare performance on the topicalized (OVS) sentences to the simple SVO sentences, and one to compare the four different question types to each other. The analysis of SVO and topicalized sentences revealed a main effect of sentence type [*F*(1,32) = 70.98, *p* < 0.001], as well as a main effect of group, showing that the hearing group outperformed the HI group [*F*(1,32) = 8.67, *p* = 0.006]. This was especially caused by lower performance on topicalized sentences as indicated by interactions of sentence type and group [*F*(1,32) = 8.39, *p* = 0.007]. One sample *t*-tests showed that the hearing group performed above chance for both conditions (*p* < 0.01), whereas the HI group performed at chance for the topicalized sentences (*p* = 0.45).

The analysis of the four question types revealed a main effect of question type (subject vs. object), object questions being overall more difficult than subject questions [*F*(1,32) = 20.84, *p* < 0.001], a main effect of Wh-phrase (*who* vs. *which*), caused by lower performance on *which* than on *who* questions [*F*(1,32) = 10.89, *p* = 0.002], and a marginally significant effect of group, caused by the HI children performing below the hearing children [*F*(1,32) = 3.79, *p* = 0.06]. There was a significant interaction of group and Wh-phrase [*F*(1,32) = 8.29, *p* = 0.007], caused by the relatively lower performance on *which* questions in the HI group. Finally, the interaction of question type (subject/object question) and Wh-phrase (which/who question) was marginally significant [*F*(1,32) = 4.03, *p* = 0.05], caused by a lower performance on *which*, compared to *who* object questions. A comparison between the two groups of the performance in each sentence type (independent *t*-tests) showed that the HI children performed significantly poorer than the hearing control group on subject *which* questions (*p* = 0.004). Their lower performance on object *which* questions differed only marginally when Bonferroni correction is applied (*p* = 0.029) from the performance of the hearing group, since some of the hearing children also still had problems with this condition.

We followed-up on the group effect and question type effect, by comparing subject *who* with object *who* and subject *which* with object *which* questions per group with paired *t*-tests. This confirmed the first impression that for each group indeed object questions were significantly more problematic than subject questions (hearing children: subject vs. object *who* questions *p* = 0.006, subject vs object *which* questions: *p* = 0.049; HI children: subject vs. object *who* questions *p* = 0.01, subject vs object *which* questions: *p* = 0.001). Furthermore, for the HI group, performance on object *which* questions was lower than that on object *who* questions (*p* < 0.006). The hearing group performed above chance on all four questions (*p* < 0.05), as indicated by one sample *t*-tests. The HI group, however, performed above chance on subject (*who* and *which*) questions and on object *who* questions (*p* < 0.05), but, importantly, they performed at chance level on object *which* questions (*p* = 0.40).

Finally, we compared the performance of each individual HI child to the hearing group (using Crawford and Howell’s *t*-test), and found that 6 of the 16 HI children performed lower than the hearing controls on the topicalized sentences and 10 of the 16 HI children performed significantly below the hearing control group on at least one question type, as shown in **Table 3**.

Interestingly, each of the six participants who performed below the hearing group on the topicalized structures was also below the hearing group on at least one type of *which* questions. Only seven HI children performed above chance on the *which* object questions, and 10 performed above chance on the *who* object questions.

Similarly to Experiment 1, these results show that some of the HI participants have problems in the comprehension of sentences that are derived by Wh-movement. Again, sentences in which the theme precedes the agent, as in topicalized sentences and in object questions seem to be especially problematic, supporting the suggestion that children with HI have a selective deficit affecting object Wh-dependencies. Object *which* questions were the most impaired type of question in the HI group.

### Overall Analysis of Difficult Structures in Comprehension in the Two Tasks According to the Individual Performance

An analysis of the two comprehension tasks that looks at the individual performance of each HI participant in each condition is also very telling with respect to the structures that are most difficult for children with HI. First, when we look at the structures in which the HI participants performed not better than chance level (at chance or below chance level, according to the binom test, *p <* 0.05), we see 4 structure in which more than 2 HI participants were no better than chance: object relatives, topicalized OVS sentences, and the two types of object questions. All these structures include Wh-movement of the theme across the agent. A second analysis, which takes into account the number of HI participants who performed below the hearing group in each condition indicates that these were also the most difficult structures according to this measure: more than 2 participants performed below the hearing group on object relatives, topicalized OVS sentences, and the two types of object questions. In this analysis, also the subject Wh-movement sentences – subject relatives and the two types of subject question – were found difficult. The two analyses are summarized in **Table 3** (shaded cells indicate the structures for which more than 2 HI children performed below the hearing group and/or at or below chance).

## Experiments 3 And 4. Repetition of Relative Clauses, Passives, Topicalization, Wh-Questions, and V-To-C Movement

After we established that some of the participants with HI had considerable difficulties in understanding sentences with Wh-movement, but not passive sentences, which are derived by A-movement, we continued to examine the various types of movement using two sentence repetition tasks.

We were mainly interested in the following comparisons: to test whether Wh-movement is impaired, we tested several types of structures that are derived by Wh-movement: relative clauses and subject- and object- *who* and *which* questions. We first tested whether these were problematic by comparing each condition to the performance on the simple SVO condition and to the performance of the hearing age-matched control group. We then compared Wh-questions that are derived by Wh-movement but keep the canonical word order of the arguments (agent before theme) and do not involve a movement of a NP across a similar NP to their non-canonical counterparts (i.e., theme before agent), that is, subject questions were compared with object questions. We further compared repetition of sentences with Wh-movement (relative clauses, Wh-questions) with sentences with A-movement (passives), with sentences in which the verb moved to second position (V-to-C movement, AVSO), and with sentences without movement (simple SVO). To test whether the existence of embedding was the source of the difficulty rather than Wh-movement, we compared sentences with Wh-movement without embedding (Wh-questions) and sentences with Wh-movement and embedding (subject relative clauses). We also compared the effect of the position of the embedded relative clause within the sentence (de Villiers et al., 1979; Correa, 1995), by comparing right-branching subject relative clauses with center-embedded subject relative clauses. Finally, we also compared long vs. short *which* questions (i.e., *which* questions with or without an extra prepositional phrase). The sentences were divided over two tasks. This way we could vary the repetition task with the other tasks and divide it over more sessions. Furthermore, the two repetition tasks differed with respect to the sentence types that were included (more details can be found in the next sessions). The two tasks will be reported separately, since the control groups that participated on the tasks are not completely the same.

### Material

#### Experiment 3: Repetition of Wh-Questions, Subject Relatives, and Passives

The sentences of the first repetition task included 10 subject questions and 10 object questions (half of each were *who* questions and half *which* questions). The *who* questions were created with an extra PP to match their length with the *which* questions; 10 passive sentences with a by phrase; and 16 subject relatives (half right-branching and half center-embedded). We also included 20 simple SVO sentences ending with a prepositional phrase as control sentences, which were included to provide a baseline as to the participants’ ability to repeat sentences without syntactic complexity, and to include some easier and less frustrating sentences for the participants. (see **Table 4** for examples).

**Table 4:** Types of sentences included in the repetition tasks.

	Wh movement	Embedding	Example
*Who* subject question with extra PP	Yes agent remains before theme	No	Wer streichelt den Igel im Käfig?
			Who_NOM_ pets the_ACC_ porcupine in-the_DAT_ cage?
*Which* subject question	Yes agent remains before theme	No	Welcher Polizist filmt den Dieb?
			Which_NOM_ policeman films the_ACC_ thief?
*Which* subject question with extra PP	Yes agent remains before theme	No	Welcher Junge berührt den Affen im Zoo?
			Which_NOM_ boy touches the_ACC_ monkey in-the_DAT_ zoo?
*Who* object question with extra PP	Yes theme moved before agent	No	Wen kitzelt der Junge am Bauch?
			Who_ACC_ tickles the_NOM_ boy at-the_DAT_ belly?
*Which* object question	Yes theme moved before agent	No	Welchen Puma beisst der Leopard?
			Which_ACC_ puma bites the_NOM_ leopard?
*Which* object question with extra PP	Yes theme moved before agent	No	Welchen Hund berührt der Junge am Kopf?
			Which_ACC_ dog touches the_NOM_ boy at-the_DAT_ head?
Subject relative right branching	Yes agent remains before theme	Yes	Das ist der Junge, der den Bäcker filmt.
			That is the_NOM_ boy, that_NOM_ the_ACC_ baker films.
Subject relative center embedded	Yes agent remains before theme	Yes	Der Tiger, der den Igel beisst, springt.
			The tiger, that_NOM_ the_ACC_ hedgehog bites, jumps.
Passive	No theme moved before agent	No	Der Tourist wurde vom Ritter gefilmt. The_NOM_ tourist was by-the_DAT_ knight filmed.
AVSO	No	No	Jetzt verfolgt der Leopard den Puma
			Now follows the_NOM_ leopard the_ACC_ puma
Simple SVO (with extra PP or Adverb)	No	No	Der Junge streichelt den Affen im Garten.
			The_NOM_ boy pets the_ACC_ monkey in-the_DAT_ garden.

#### Experiment 4: Repetition of Wh-Questions, and V-to-C Movement Derived Sentences

The second repetition task consisted of long subject and object *who* and *which* questions (5 each, with an extra PP for all four questions types), and simple canonical sentences that started with an adverbial phrase, and hence included the verb in second sentential position, before the subject (AVSO, 10 items), or ended with adverbial phrase (SVOA, also 10 items).

The sentences of the various types, 132 in total for the two tasks^8^, were presented in random order, in smaller blocks of 20–40 sentences, sometimes with several blocks per session over at least two sessions (for some children more sessions were needed, with a maximum of five sessions in total). All sentences were semantically reversible and included a transitive verb. In the center-embedding relative clauses, the matrix verbs were intransitive and the embedded verbs were transitive. Apart from the SVOA and AVSO sentences, the two NPs were of masculine gender in all sentences, to preclude (temporary) structural ambiguity (as in German only masculine determiners distinguish between nominative and accusative case). Since structural ambiguity was less of a problem in AVSO sentences and in order to create more variation in the material, in 5 of the SVOA and 5 of the AVSO sentences one NP was feminine or neuter.

All sentences consisted of 5 to 8 words, a perfect matching with respect to number of words was not possible. However, whenever there was an unavoidable difference, we made sure that sentences we expected to be relatively easier were longer than sentences that were expected to be relatively more complex instead of vice versa. So, e.g., the supposedly easier right-branching subject-relatives consisted of eight words (the only 8-word condition), whereas the syntactically more complex center-embedded subject-relatives consisted of six words.

### Procedure Experiments 3 and 4. Sentence Repetition

The experimenter read a sentence in a relaxed pace and in a normal (neutral) intonation meaning that she did not use a specific focus intonation for object-first sentences, for instance, but questions were consistently produced with a question intonation. The participant was requested to count to 3 out loud and then to repeat the sentence as accurately as possible.

The counting was used to prevent rehearsal in the phonological loop (Baddeley, 1997; Friedmann and Grodzinsky, 1997), and hence to preclude phonological echoing. The whole session was audio-recorded and afterward transcribed for further analysis.

### Error Analysis Experiments 3 and 4. Sentence Repetition

In the analysis of errors in repetition, structural errors were scored separately from lexical and morphological errors that did not affect the structure and the thematic roles in the sentence. Phonological errors and other errors resulting from articulatory problems in which the target words and structure were still recognizable were ignored.

An error was classified as a **structural error** (see examples in 12), when the child changed the structure of the sentence, changed the thematic roles in it, or produced an ungrammatical sentence, for instance by using the same case twice (resulting in a sentence with two nominatives or two accusatives). **Lexical errors** were errors that included substitutions of a NP with another NP that did not appear in the target sentence (a singer → a dancer), a substitution of the verb with another verb with the same argument structure (like → love), and a few omissions or additions of the definite article (the elephant → elephant), or a, substitution, or addition of the adverbial or prepositional phrase (yesterday → today).

(12) Examples of **structural errors** for target sentence:

                               Welchen Puma beisst der Leopard?’

                               Which_ACC_ Puma bites the_NOM_ leopard?

         Role reversal with a structure change (object questions > subject question):

                               Welch**er** Puma beisst d**en** Leopard

                               Which_NOM_ Puma bites the_ACC_ leopard?

         Role reversal without structure change (Noun reversal)

                               Welchen **Leopard** beisst der **Puma**?

                               Which_ACC_ Puma bites the_NOM_ leopard?

         Noun doubling (one of the arguments receives both roles):

                               Welchen Puma beisst der **Puma**?

                               Which_ACC_ Puma bites the_NOM_ Puma?

         Case error (two nominatives):

                               Welch**er** Puma beisst der Leopard?

                               Which_NOM_ Puma bites the_NOM_ leopard?

As can be seen in (12), some lexical substitutions were indicative of a problem with the thematic roles of the sentence, and were hence counted as structural errors. These included substitution of one of the NPs in the sentence with the other NP, i.e., in noun doubling, yielding a sentence in which one of the NPs appears on both roles (*which puma does the leopard bite* →* which puma does the puma bite*), and reversals (→*which leopard does the puma bite?*).

Finally, **morphological errors** that did not affect the thematic grid of the sentence and did not pertain to the syntactic structure, were counted separately from the structural errors and grouped together with lexical errors. These were mainly gender errors or number errors (changing a singular NP into a plural), and some instances of an accusative that was changed into a dative case (Wem beisst der Leopard → who_DAT_ does the leopard bite). This latter error type was the only case error that did not count as structural error, since a confusion of accusative and dative in our task did not affect the overall structure, and crucially did not affect the assignment of either syntactic or semantic roles, since both are clearly objective case.

## Results: Repetition of Subject Relatives, Wh-Questions, Passives Topicalization, and V-To-C Movement Derived Sentences

### Experiment 3: Repetition of Passives, Topicalization, and Wh-Questions

**FIGURE 5:**
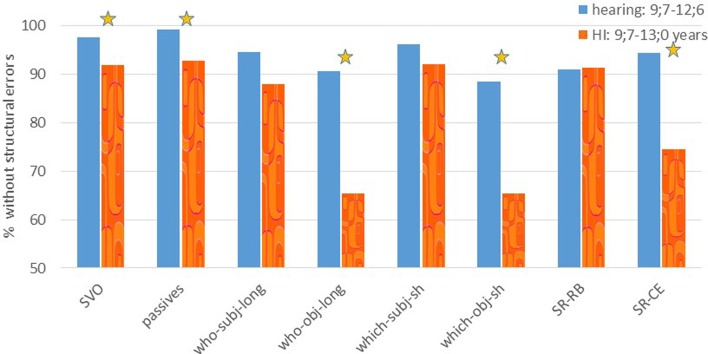
**Experiment 3: % of sentences repeated without structural errors**. SVO, passives, long subject and object *who* questions, short subject and object *which* questions, SR-RB: right-branching subject relatives and SR-CE: center embedded subject relatives. A star indicates a significant difference between the groups.

**FIGURE 6:**
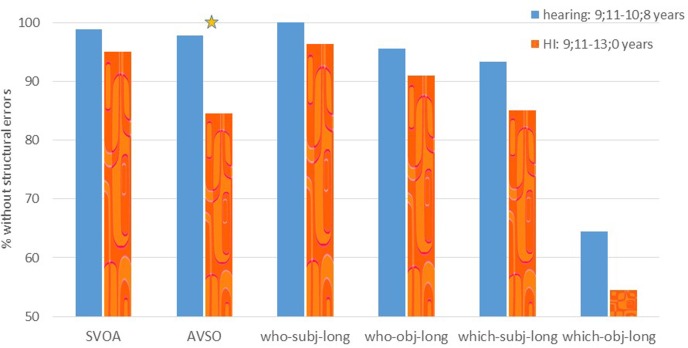
**Experiment 4: % of sentences repeated without structural errors**. The star indicates a significant difference between the groups.

The results presented in **Figures 5** and **6** and in the analysis below include only structural errors, whereas sentences that were repeated only with lexical and/ or morphological errors were scored as correct repetitions for this analysis.

**Figure 5** shows the results of the first sentence repetition task. This task included simple SVO sentences (with an extra PP to match for number of words), passives (with a by phrase), right-branching and center embedded subject relatives as well as short subject and object *who* and *which* questions. This task was performed by 15 HI children (age 9;7–13;0, mean 10;8), and 47 age-matched hearing children (age 9;7–12;6; mean 10;10).

To analyze these data, we first ran a repeated measures test with group (hearing vs. HI) and sentence type as variables. This revealed a main effect of sentence type [*F*(7,420) = 14.42, *p* < 0.001], and a main effect of group [*F*(1,60) = 12.59, *p* = 0.001]. Also an interaction of sentence type and group was found [*F*(7,420) = 6.09, *p* < 0.001]. To follow this up, we compared the performance of the two groups on each sentence type. This revealed that the HI group performed significantly worse on SVO sentences, *who* and *which* object questions and center-embedded subject relatives (*t*-tests, *p* < 0.05, see **Table 5**). No difference between groups was found for the *who* and *which* subject questions and the right-branching subject relatives.

**Table 5:** Comparison of sentence repetition without structural errors in the HI and hearing groups per sentence type.

Structure	Comparison between HI and hearing groups
Simple SVO sentence	*t*(60) = 2.68	*p* = 0.009^∗^
Passive sentence	*t*(60) = 2.25	*p* = 0.03
*Who* subject question	*t*(60) = 1.50	*p* = 0.14
*Who* object question	*t*(60) = 4.06	*p* < 0.001^∗^
*Which* subject question	*t*(60) = 1.14	*p* = 0.26
*Which* object question	*t*(60) = 3.23	*p* = 0.002^∗^
Right branching subject relative	*t*(60) = -0.07	*p* = 0.94
Center embedded subject relative	*t*(60) = 3.58	*p* = 0.001^∗^

*^∗^The HI group performed significantly below the hearing group, in an independent *t*-test using FDR correction for multiple comparisons (Benjamini and Hochberg, 1995)*.

Finally, we ran repeated measures per group with *post hoc* pairwise comparisons (Bonferroni corrected) to see which conditions were most problematic in each group. This revealed a significant main effect of sentence type for the hearing children [*F*(7,322) = 3.79, *p* = 0.001]. Pairwise comparisons showed that passive sentences were significantly easier than object *who* and *which* questions (*p* < 0.05). For the HI group, we also found a main effect of sentence type [*F*(7,98) = 10.41, *p* < 0.001]. Pairwise comparisons revealed that SVO, passive sentences, and subject *who* questions, as well as right-branching subject relatives were repeated better than object *who* and *which* questions (all comparisons *p* < 0.05, Bonferroni corrected, see Appendix B).

An analysis of the performance of each individual HI participant compared with the hearing group revealed that object questions were difficult also at the individual level, and were more difficult than the parallel subject questions. As summarized in **Table 6**, the structures on which the performance of the HI children was most deviant from that of the control group, namely, on which there were more HI children who performed below the aged-matched hearing children, were object *who* questions, where 7 HI children had a lower performance than the hearing children and object *which* questions, where 5 HI children were below the controls (there were fewer HI children below the controls on the parallel subject *who* and *which* questions). The below-control performance of some HI children on SVO and passive sentences, probably resulted from the ceiling performance of the hearing children, which made a single error already significantly below the hearing group. There was considerable overlap between the HI children who performed significantly below the controls in the various constructions: seven HI children performed below the hearing controls on at least two conditions, (4 of them on 4 and more conditions), and only three showed impaired performance on only one condition (one of them was very close to the cut-off point in three additional conditions, so he was probably impaired, and one only made a single error in the SVO condition, which qualified as significantly below the control, but he was probably unimpaired).

**Table 6:** Repetition Experiments 3 and 4: number of HI participants performing significantly below the hearing group.

	Repetition 1 (no. out of 15 participants)								Repetition 2 (no. out of 11 participants)					
	SVO	Passive	Subject who	Object Who	Subject Which	Object Which	Subject relatives RB	Subject relatives CE	SVOA	ASVO	Subject *who*	Object *who*	Subject *which*	Object *which*
No of HI below hearing group	3	2	3	7	1	5	1	4	2	5	1	2	3	3

*Comparison to the control group using Crawford and Howell’s *t*-test, all *p* < 0.05*.

#### Error Analysis Experiment 3

As can be seen in **Table 7**, most of the structural errors that the children made relate to syntactic/semantic role assignment. The HI children made many case errors when they tried to repeat Wh-questions. These errors resulted in an ungrammatical sentence with either two nominatives or two accusatives. Importantly, such errors occurred almost exclusively when the HI children tried to repeat an object question, and not when they tried to repeat a subject question. Other errors relating to the syntactic/semantic roles are head doublings or reversals, as well as canonization, which means that in repeating an object-first sentence, a child produces a grammatical (but non-target) subject-first sentence. Interestingly, some of the errors on the center-embedded subject relatives are changes into right-branching subject relatives (these are the word order errors in the center-embedded subject relatives in **Table 5**). The few word order errors that occurred in the canonical-order sentences (SVO, subject questions, and right-branching subject relatives), 5 errors in total in these structures, were object-first sentences. Errors in the Wh-word consisted of omission of the Wh-word and use of full NP instead, or use of *who* instead of *which* or vice versa. Other errors consisted of omissions of one of the DPs, fragments, or in subject relatives: omission of one of the verbs.

**Table 7:** Experiment 3- structural errors in repetition: number of errors per sentence type.

			*Who* questions		*Which* questions		Subject relatives	
Error types	SVO	Passives	Subject	Object	Subject	Object	RB	CE
Canonization		1		2		6		
Noun doubling/reversal	6	5				3	2	
Case error	2	1	1	14		12	3	
Word order	2				1		3	6
Wh-word			2	2	1	2		
Other	10	5	4	3	4	2	2	17

To summarize, HI children performed worse on the repetition task than the hearing children. Interestingly, as we saw also in the comprehension studies, not all movement-derived sentences were equally problematic. Passive sentences caused relatively little problems, and the performance of the HI group in repeating them was very similar to their repetition of simple SVO sentences, although it has to be acknowledged that there was a group difference for the SVO sentences, which can be explained by the ceiling performance of the hearing children (as we argued above). In contrast, object questions, which are derived by Wh-movement, were especially difficult. These problems seem to be caused by the fact that in object questions the theme is moved over the agent of the sentence. Subject *who* and *which* questions, which involve Wh-movement but in which the theme follows the agent, did not cause repetition problems for the HI group. Furthermore, most errors on the object questions were related to syntactic/semantic role assignment. Finally, center embedded subject relatives, but not right-branching subject relatives, are problematic for the HI children.

### Experiment 4: Results Repetition of Wh-Questions and V-to-V Movement Derived Sentences

The results of the second sentence repetition task, which compared Wh-questions (long subject and object *who* and *which* questions) and AVSO sentences to simple sentences, are presented in **Figure 6**. This task was performed by 11 HI children (age 9;11–13;0, mean 11;0), and 9 hearing children, in the age of the youngest HI participants (age 9;11–10;8, mean 10;3).

We ran two separate repeated measures, one to examine verb movement, by comparing the performance on SVOA and AVSO sentences, and one to examine Wh-questions, by comparing the four different question types to each other (with two variables: question type- subject or object question, and Wh-phrase – *who* or *which*). The verb-movement analysis revealed a significant effect of sentence type [*F*(1,18) = 13.14, *p* = 0.002], an interaction of group and sentence type [*F*(1,18) = 8.73, *p* = 0.008], and a marginally significant difference between the two groups, caused by overall lower performance of the HI children [*F*(1,18) = 4.27, *p* = 0.054]. This was caused by a lower performance on the AVSO sentences, but not on the SVOA sentence in the HI group (as indicated by *post hoc* independent *t*-tests, SVOA: *t*(18) = 1.05, *p* = 0.31, and AVSO: *t*(18) = 2.54, *p* = 0.02, respectively). The analysis of the Wh-questions resulted in a main effect of question type, with subject questions repeated correctly significantly more often than object questions [*F*(1,18) = 18.77, *p* < 0.001]. A main effect of Wh-phrase was also found, caused by significantly more correct repetitions for *who* than for *which* questions [*F*(1,18) = 15.89, *p* < 0.001], as well as an interaction of question type and Wh-phrase, caused by relatively fewer correct repetitions for object *which* questions [*F*(1,18) = 11.62, *p* = 0.003]. No main effect of group and no interactions with group were found.

The comparison of the performance of each HI individual with the hearing group is summarized in **Table 6**. It indicates that 2 of the 11 HI children performed below the hearing control group on the SVOA sentences, and 5 were below the hearing group on AVSO sentences; 3 children performed below the hearing children on the *which* object questions, 3 on the *which* subject questions; 2 on the *who* object questions and one of the *who* subject questions (all *p* < 0.05, Crawford and Howell’s *t*-test). The children who performed significantly lower on many of the conditions (6 or 7) of the first repetition task, performed poorly also in this task.

#### Error Analysis Experiment 4

The error analysis on the second repetition task (see **Table 8**) revealed that most errors on object questions can again be connected to problems with syntactic/semantic role assignment: canonization errors (changing an object question to a subject question), case errors, and noun doublings or reversals. The canonization error of the AVSO sentence involved a change into an SVO sentence. Errors with the Wh-word consist of omission of the Wh-word and use of full NP instead, or use of *who* instead of *which* or vice versa. Other errors consisted of omission of one of the arguments, the verb, or an otherwise fragmentary response.

**Table 8:** Experiment 4 – structural errors in repetition: number of errors per sentence type.

			*Who* questions		*Which* questions	
	SVOA	AVSO	Subject	Object	Subject	Object
Canonization		1				15
Noun doubling/reversal	1	2		3	2	1
Case error	2	4	1	4	2	1
Wh-word			1		1	1
Other	3	7			3	6

This sentence repetition task, like the previous repetition task, and similarly to the results of the comprehension task indicated that the children with HI had difficulties in structures that are derived by Wh-movement, especially when the theme precedes the agent, i.e., in object questions. *Which* questions were more problematic than *who* questions. Whereas the HI children repeated sentences that involved a movement of the verb to second position (AVSO sentences) less well than the hearing children, their performance on the AVSO sentences was still better than their performance on the object questions.

Experiment 4 showed partially different results than Experiment 3, in that the hearing children did not perform very well on the object *which* questions yet. This may have been caused by the fact that the hearing children in this task were overall younger (up to age 10;8) than the children who participated in Experiment 3 (up to age 12;6). This, combined with the fact that the *which* object questions in Experiment 4 were slightly longer (since we had added a prepositional phrase), may have caused their lower performance, which then resulted in the absence of the interaction.

Nevertheless, the findings of the repetition tasks join those of the comprehension tasks in indicating that the HI children show a selective deficit affecting object Wh-dependencies.

## Individual Performance Patterns in All Four Tasks

The comparison of each individual HI participant to the hearing group (as tested with the Crawford *t*-tests) for the two comprehension and repetition tasks revealed that almost all HI children had problems in at least some comprehension or repetition of movement derived sentences. We classified the children according to the comparison of each of them to the control group, in children with good performance, almost good performance, mild impairment, or severe impairment.

Children with normal performance performed below the hearing group on one condition at most, and with a maximum of 2 errors on this condition, indicating that performance was still close to the normal hearing performance. Children with almost-normal performance performed below the hearing group on 2 of the movement conditions, and performed well (above chance performance) on all the other conditions. Children with a mild impairment performed clearly below the hearing group on one or two conditions, and performed at (or below) chance on this and/or at two other conditions. The severely impaired group performed significantly below the hearing group for at least six conditions (for those children who performed all tasks; or three conditions for the children who performed only two of the four tasks).

This way, our group of HI children consisted of only three HI children whose syntactic performance was within the normal range (participants 1, 8, and 9), and one HI child with near-normal performance (23). The rest 14 HI children had a syntactic impairment: Six HI children (2, 3, 4, 6, 7, and 18) who were tested on both repetition and comprehension were severely impaired in several conditions and the four children that were only tested on comprehension (11, 12, 16, and 17) were all impaired on at least three conditions. Five additional participants had a mild syntactic impairment (5, 21, 22, 24, and 26).

Furthermore, a Guttman Scale (Guttman, 1944, 1950)^9^ was found in the comprehension of Wh-movement and passives, suggesting ranking of impairment of the two structures: the two children who failed to understand passives also had considerable problems in understanding object Wh-movement sentences (i.e., object relatives, topicalized sentences, and object questions), and there were children who failed only on Wh-movement derived sentences, but not on passives. That is: there were no children who failed on passives but had no problems with object Wh-movement. These results show that not all types of movement result in the same difficulty in HI children, and that a deficit in passives is more severe than a deficit in Wh-movement alone, and involves a deficit in Wh-movement as well.

Finally, an analysis of the background of the subgroup of the 4 children who showed normal or near-normal syntax either received hearing- aids during the first year of life (participant 9) or received hearing aids after age 5 years (participants 1, 8, 23), and there is no information that their hearing was impaired earlier, and therefore they may have been hearing normally during the first year of life and lost their hearing only at a later age. This suggests the pivotal role of early language exposure in later development of syntactic abilities. It would be very interesting to see, in future research with a larger HI group with more detailed background data, if input during the first year of life correlates with later syntactic performance.

## Discussion

The aim of this study was to examine whether lack of sufficient exposure to language from birth affects German-speaking children with HI in their comprehension and repetition of sentences that are derived by syntactic movement. Our second aim was to compare three types of syntactic movement: Wh-movement, passives (A-movement), and verb movement (as in V-to-C movement). One of the reasons that make this study in German especially interesting is that it allowed us to examine whether German-speaking HI children can use case morphology for the correct interpretation and repetition of these movement-derived non-canonical sentences. German furthermore allowed us the direct comparison of different types of syntactic movement.

Our results indicated that most of the children with HI showed considerable difficulties in both sentence repetition and comprehension, and performed significantly poorer than hearing children. Importantly, their difficulty was selective and did not span over all sentence types. The comprehension of the HI children was significantly lower than that of the hearing group in subject and object relatives, topicalized sentences (OVS) and object *who* and *which* questions. In contrast, they performed similarly to the hearing children on simple SVO sentences, passives, and on subject *who* and subject *which* questions. These structures were also the problematic ones according to the individual-level analysis of the number of HI children who performed worse than the controls and the number of HI children who performed not better than chance level. This indicates that it is not any type of syntactic movement that results in comprehension deficits, but specifically Wh-movement that is the problem.

The sentence repetition of the HI group showed a similar selective impairment. Their repetition of object *who* and object *which* questions, center-embedded subject relatives and AVSO sentences showed considerable impairment and resulted in performance that was significantly poorer than that of the hearing children, at the group level, and for most of the HI participants also at the individual level. In contrast, performance on subject *who* and subject *which* questions, as well as on simple SVOA sentences and right-branching subject relatives did not differ from their age-matched hearing group (object relatives and topicalized sentences were not reported for the repetition tasks, due to the low performance even in the hearing group).

It has to be noted though, that overall there was much variation in the HI group, as became clear by the analyses in which we compared the performance of each HI participant to the hearing group. Only three of the 19 HI children performed just like the hearing children. Most HI children quite clearly performed (much) poorer than their age-matched peers on more than one condition that involved syntactic movement. Some HI children even performed poorer than hearing children on the syntactically less complex conditions (e.g., passives, subject questions, or even on SVO, see **Table 3**). We will discuss these results in detail below, where we start with a discussion of the comprehension and repetition of the three different types of syntactic movement: Wh-movement, A-movement, and verb movement. Then we will compare the results on these structures, and finally we will discuss possible explanations for the variation in performance.

The poor performance that our German-speaking HI participants demonstrated in the comprehension and repetition of structures that are derived by Wh-movement of the object is in line with previous studies on HI syntax in English, Hebrew, Arabic, and Italian. Object relatives, object topicalized sentences, and object questions are all sentences that are derived by Wh-movement, in which the theme moves to a position before the agent, as explained in the introduction, and various studies demonstrated that children with HI are impaired in such structures (Quigley et al., 1974a,b; Geers and Moog, 1978; Berent, 1988, 1996; de Villiers, 1988; de Villiers et al., 1994; Friedmann and Szterman, 2006, 2011; Friedmann et al., 2010b; Friedmann and Haddad-Hanna, 2014; Szterman and Friedmann, 2014b, 2015; Volpato and Vernice, 2014). The poorer performance in center-embedded relative clauses compared with right-branching^10^ ones is also in line with previous literature (see e.g., Quigley et al., 1974a; de Villiers et al., 1979, where the performance on right-branching object relatives was better than on center-embedded object relatives for hearing children).

Thus, sentences derived by Wh-movement seem to be especially impaired, in both comprehension and repetition, especially those sentences in which the theme moved to precede the agent of the sentence. German also allowed us to examine two further types of movement: A-movement, which occurs in passive sentences, and verb movement to second position. The results indicated that the deficit of the HI children did not extend to all types of syntactic movement. Starting with passive sentences, which are derived by A-movement, a type of movement that is shorter than Wh-movement (from object position to subject position, roughly), our findings indicate that they did not show the same impairment as did the sentences derived by Wh-movement: only 2 children failed to understand the passive sentences, and in fact, the performance on the comprehension of the passive sentences was just like the performance on the simple SVO sentences. These findings indicate that different types of movement are impaired differently in HI, and that not all types of movement are impaired in HI. It moreover shows that the impairment is not merely a problem in non-canonical sentences. In passives too the theme comes before the agent, yet, most HI children do not have problems comprehending and repeating those structures. Note that the few HI children that do have problems in passives, always also have problems with Wh-movement, which does not hold the other way around. It seems to be the case that Wh-movement is impaired, especially so when the theme has moved over the agent, whereas A-movement (as seen in passives) seems to be relatively well-comprehended by most HI children.

The relatively good comprehension of passives in German is in contrast to findings in earlier studies on English (Power and Quigley, 1973; Nolen and Wilbur, 1985; Schmitt, 1968), where the comprehension of passives was reported to be impaired. One explanation for this difference could be that the children in these studies had a more severe HI than our children. At least for the Power and Quigley and the Nolen and Wilbur data this seems to be the relevant difference between their and our participants.

Can this difference between English and German be ascribed to the fact that in German case-marking can indicate the agent and the theme in the sentence? Definitely not: in passive sentences the theme is actually marked as the subject of the sentence, and hence, if anything, the case-marking is liable to confuse the children, unlike in the Wh-movement structures. But in effect, the picture that emerged from the HI performance was the opposite: they succeeded in passives and failed in Wh-movement, so it cannot be case-marking that saved their interpretation and identification of agent and theme.

Interestingly, other populations have been found to show similar difficulties in the comprehension and/or production of complex syntactic structures. People with agrammatic aphasia for instance show a severe deficit in the production of relative clauses, Wh-questions, and embedded structures (Friedmann, 2001, 2006; Ruigendijk et al., 2004), as well as in the comprehension of object Wh-questions, object relative clauses, topicalization structures, and (for some patients) passive sentences (see, among many others, Zurif and Caramazza, 1976; Grodzinsky, 2000; Friedmann and Shapiro, 2003). Children with a Specific Language Impairment (SLI), specifically children with syntactic SLI, show a significant specific deficit in the comprehension and production of sentences with movement dependencies such Wh-questions and relative clauses (e.g., van der Lely, 1998; Bishop et al., 2000; Friedmann and Novogrodsky, 2004, 2011; Novogrodsky and Friedmann, 2006). Whereas the deficits in the three populations seem similar, it might still be that the underlying psycholinguistic and neural bases of the syntactic impairment is different in each of these populations. Szterman and Friedmann (2014b) in fact suggested that the HI population includes (at least) two patterns of impairment, one characterized by impairment in the CP layer of the syntactic tree, similar to theories regarding agrammatic aphasia (Friedmann and Grodzinsky, 2000; Friedmann, 2006), whereas other HI children show a deficit in movement that is more similar to the one evinced in syntactic SLI. A further question is whether the syntactic problems in HI children should be characterized as a deficit, or as a delay in development. This question cannot easily be answered on the basis of our data. However, the fact that some of our HI children who performed well below the hearing group were 11 years and older, this may be an indication for a more persistent impairment. In other studies (e.g., Friedmann and Haddad-Hanna, 2014), even 21 year old HI participants demonstrated the same types of syntactic deficits, suggesting that at least in some cases the syntactic deficit is a deficit rather than a delay. An interesting approach to the term “delay” may be the following: we may thing of HI individuals as having a syntax that has been “stuck” at some stage of normal development.

Importantly, case did not seem to assist the participants in their interpretation of the object relatives, object questions, or topicalized OVS sentences: in all these sentence types, the agent is marked with a nominative case and the theme with an accusative case. Nevertheless, these were the structures that the participants found most difficult. Therefore, we can conclude that they could not utilize the case markers to assign the thematic roles in the sentence (see Friedmann et al., 2017, for a related discussion). In fact, 10 of the HI children performed below chance level, consistently reversing the roles of the agent and the theme in at least one of the Wh-movement sentence conditions. This indicates that not only do these children not use case for interpretation; they even do not take it into the computation of thematic roles at all, and ascribe roles as if case did not exist in the sentence, on the basis of the linear order of the two NPs. Importantly, their inability to use case for interpretation is not a result of them not being able to hear the case markers: we were very careful to only include in the study children who performed well in the auditory discrimination task that included phrases with determiners and Wh-elements marked for nominative and accusative case (see General Method).

It is possible that what makes passive sentences easier for the HI children than sentences with object Wh-movement is the passive auxiliary (*wird* or *wurde*) and the by phrase (*vom*), which provides a signal beyond case that the sentence is not a simple SVO sentence.

The error types in the repetition task provide further support for the specific problems in sentences that are derived by Wh-movement of the theme across the agent, most errors somehow relate to the semantic/syntactic role assignment. Either the sentence is canonized, that is, an object first structure is changed into a subject first structure, or, the NPs are reversed. Also frequently occurring for the object structures, is a case error, for instance repeating the sentence with two nominatives or accusatives, suggesting that the child starts out with a nominative NP, then seems to realize the final NP was a nominative, or vice versa: s/he starts repeating the first NP correctly as an accusative and then in between ‘canonizes’ and ends up with a second accusative NP. The use of case markers, even when they map the thematic roles incorrectly, adds support for our conclusion that even though HI children cannot use case markers for comprehension, they do hear them, store them, and know their morphological distribution.

Interestingly, object *which* questions were the most impaired type of question in the HI group, both in sentence comprehension, and in the second repetition task^11^. These object *which* questions seemed to cause similar comprehension problems as object relatives and topicalized sentences. The difference between object *which* questions and object *who* questions has been reported before, for HI children (Friedmann and Szterman, 2011), and also for other populations such as young hearing children (De Vincenzi et al., 1999; Avrutin, 2000; Friedmann et al., 2009; Biran and Ruigendijk, 2015; and children with S-SLI, Friedmann and Novogrodsky, 2011). It has been explained by the fact that in *which*, but not in *who* questions there are two lexical NPs (which NP and the subject NP), whereas in object *who* questions, there is only one lexical NP (the object NP), and a *who* phrase. The argumentation is, that when the moved element (here: the object) is similar in structure to the element it moves over (here: the subject), then the structure is more problematic in child language than if the moved element is less similar. In a *which* question, a full NP (welchen NP, ‘which_ACC_ NP’) moves over the subject to the first position of the sentence, whereas in an object *who* question, only a Wh-phrase moves (wen, ‘who_ACC_’). Similarly to *which* questions, also in topicalized sentences and in object relatives a full lexical NPs moves over the subject NP, which may explain the similar performance on these three conditions (see Friedmann et al., 2009; Belletti et al., 2012; Biran and Ruigendijk, 2015 for a more detailed account)^12^. Apparently, what is difficult in normal language acquisition, is even more difficult in acquisition for HI children.

Furthermore, the comprehension of subject relatives, as well as subject *who* and *which* questions overall was less problematic. That is when comparing the two groups, sentences with Wh-movement in which the theme did not cross the agent caused less comprehension problems than sentences with crossing of the theme over the agent. Nevertheless, the individual results show that these structures too caused some difficulties for some HI children. The problems of these four participants with subject Wh-movement sentences were most pronounced in the repetition task.

At least for some of the children, the reason for this pattern, which shows good performance on sentences with Wh-movement in which the agent remains before the theme, and poorer performance on the repetition of these structures may be related to an impairment in the syntactic tree. Szterman and Friedmann (2014b) found that whereas the syntactic deficit of many of their HI participants was a deficit in Wh-movement, there were some children whose syntactic deficit was of a different sort: they had a deficit in the highest node of the syntactic tree, CP (similar to the impairment in agrammatism, see Friedmann, 2001, 2006; and to de Villiers et al., 1994 suggestion for all HI children). In German, every sentence that involves Wh-movement requires lexical items to reach the CP layer^13^. As a result, all Wh-movement sentences, should be difficult to produce for individuals with CP impairment, both those in which the agent has moved (subject relatives, subject questions), and those in which the theme moved. In a sentence-picture matching comprehension task, a strategy that ascribed the first NP the agent role can still guide the participant to point to the correct picture in Wh-movement sentences in which the agent moved and remained before the theme. However, object Wh-movement sentences would show impairment in such tasks. The story is different in repetition: here, an agent-first strategy cannot salvage sentence repetition, so the difficulty would manifest itself also in the repetition of subject relatives and subject questions. Supporting this view is the fact that all the children who failed to repeat subject Wh-movement (and not just object Wh-movement) also failed to repeat AVSO sentences, in which the verb moves to the CP layer (one of this children was 0.01 points above the threshold for the verb-movement structures). Those five children that performed worse on the AVSO than the hearing group were even more impaired on the object which questions, which would be in line with the idea that these children not only have a problem in Wh-movement, but also in using the CP layer.

Finally, whereas some HI children had severe syntactic difficulties, others performed much better in both comprehension and repetition. A possible explanation may be found in the age of implantation and/or the age of hearing loss of the children who showed better syntactic abilities. Of the 4 children who showed age-appropriate or near-normal syntax in our tasks, one received hearing devices at a very young age (at or before age 1;0), pointing, very carefully to the importance of exposure to language during the first year of life. The three other HI participants were diagnosed with a HI quite late (5;0, 6;0, and 8;0) which may indicate that the hearing loss was not present from birth, so that they were actually exposed to language normally during the first year of life.

One interesting question is what exactly it is in the early exposure to language that is needed to acquire syntactic structures derived by Wh-movement. One possibility is that the phonological properties of the structures we tested are especially difficult for a hearing-impaired child to perceive during the early critical period: e.g., the German case markers or the prosody of topicalized sentences. However, this does not seem to be the case: the specific difficulty in Wh-movement structures is typical also to young hearing TD children and to hearing children with syntactic SLI (e.g., Friedmann and Novogrodsky, 2004, 2011; Friedmann et al., 2009; Biran and Ruigendijk, 2015). Additionally, whereas the perception of the case markings on the German determiners may be difficult, difficulties in parallel sentence structures are also apparent in languages in which topicalization and relative clauses are marked by word order and not by phonologically similar case marking on determiners, such as Italian, English, and Hebrew. Therefore, it does not seem to be difficulty in hearing specific parts of the sentence in early childhood that hampers the acquisition of Wh-movement structures, but rather something more general about exposure to language in the first year. It is currently an open and especially intriguing question of what exactly is the type of language input that is required during the critical period for Wh-movement.

Another possible account would ascribe the syntactic difficulty of HI children in specific structures to their difficulty with respect to, for example, perceiving the different case morphemes, despite normal syntactic abilities. The results, however, are not consistent with this approach either: good syntactic abilities with poor perception would end up in repeating sentences possibly with incorrect case relative to the target sentence, but the repeated sentences are then expected to be grammatical. Such an approach cannot account for the error pattern that our participants exhibited in sentence repetition, where, for example, they produced sentences with the same case twice.

We have admittedly a very small sample of participants and hence our data can only be taken as a possible indication for future research. Nevertheless, these results are consistent with similar reports from larger groups of HI in Hebrew (Friedmann and Szterman, 2006; Szterman and Friedmann, 2014b, 2015), and Arabic (Friedmann and Haddad-Hanna, 2014), where the HI children who succeeded in syntactic tests were the ones who received hearing aids before the age of one year. Therefore, we may suggest that although early implantation or aiding does not guarantee good syntactic performance later on, as some HI children who were aided at a very young age still had considerable syntactic problems, early exposure to language input emerges as a necessary condition for the normal development of syntactic abilities.

## Ethics Statement

This study was approved by Kommission für Forschungsfolgenabschätzung und Ethik der Universität Oldenburg. We informed all parents of our study, the methodology, and procedure. Only children whose parents gave informed consent (written, with signature) took part in this study. Furthermore, only children who wanted to participate, participated, and they could stop their participation at any moment when they wanted, without further consequences. Both parents and children were explicitly informed about this.

## Author Contributions

ER and NF created together the research question and design. They adapted the Hebrew tests together to German. ER ran the tests and coded the results. ER and NF analyzed and interpreted the results together, wrote the paper together, and each did part of the statistical analysis.

## Conflict of Interest Statement

The authors declare that the research was conducted in the absence of any commercial or financial relationships that could be construed as a potential conflict of interest.

## Footnotes

1 Note that only for masculine NPs nominative and accusative are unambiguous (der vs den); for neuter (das), feminine (die), and plural (die) definite articles, nominative, and accusative are the same. Unless stated otherwise, we use and discuss the unambiguous case marker in this paper.

2 We abstract away here from movement of the verb from its VP-internal (verb phrase-internal) position. It is argued that in German, the verb is base generated at the final position, hence canonical order would be SOV. In these examples the original position of the verb does not affect the assignment of the thematic roles.

3 NOM refers to nominative case, usually used for the subject of the sentence, ACC refers to accusative case, used for –among other categories- the object of a sentence, and DAT refers to dative case, which is used –among other things- after some prepositions, like the P *von* ‘by’ in passive sentences (like 10).

4 In addition to morphosyntactic information, the different structures may be argued to be realized with different prosody. Weber et al. (2006) for instance conclude that prosody can influence the interpretation of constituent order ambiguities (possible in German, see footnote 2) in that a prosodic manipulation (i.e., marked prosody with narrow focus on the first NP) eliminated the normally existing subject-first preference. Importantly though, the prosodic information did not make an object-first interpretation more preferable, showing that prosody alone is not enough for disambiguation of these structures, whereas morphosyntactic information can be. Similarly, Braun (2006) provided experimental evidence for a different prosody for topicalized sentences in a production task in German, but again this prosodic information could not be reliably used for comprehension (see also Pappert and Pechmann, 2012, or Carroll, 2013 for a discussion).

5 In cases of difficulties in the CP-layer of the syntactic tree, one may leave the subject in spec-IP (inflectional phrase) and the verb in I, or even leave the subject and the verb VP-internally. An SV sentence may still sound exactly the same as an SV sentence in which the S moved to spec-CP and the verb to C and is hence not a very good way to test verb-movement.

6 One additional task, elicited production, was tested in these sessions as well, contributing to the overall variation in tasks. Data from this task will not be reported in this paper, because even the hearing participants in the ages we tested still do not master the production of object relatives, so we could not use this task to compare the abilities of the HI to the hearing children.

7 For some children, not all items could be tested because they were either too tired, or for organizational reasons.

8 Three additional conditions (topicalized sentences and two types of object relatives) with a total of 26 items were initially included in the task. These will not be reported here, because even some of the 11-year old hearing children still made errors in repeating them.

9 Louis Guttman initially suggested this approach for establishing a scale on dichotomous assertions. The idea was to examine whether items where a person either endorses or does not endorse a statement form a scale. If these statements form a scale, then a ranking of statements is possible, in which a person who endorses a certain statement, would also endorse all statements that are ranked lower in the scale. We use this approach to examine whether the impairment on the various syntactic structures forms a scale for the population of hearing impaired children, using whether or not a person succeeded in a certain structure as the dichotomous measure.

10 Both right-branching and center-embedded subject relatives show a form of Wh-movement and are hence in principle equally difficult, but some factor made the participants repeat the right-branching relatives better. One possibility is that whereas the comprehension of right branching subject relatives can benefit from a strategy according to which the first DP is the agent (Grodzinsky, 1995), for a center-embedded relative this would not be enough. If the trace of movement is not identified correctly, the main verb might not be identified as such, and the identification of its argument might become difficult, leading to impaired repetition.

11 Note that the hearing children had a relatively low performance on object *which* questions in this task as well and therefore no interaction with group was found here. Most likely this was caused by the fact that this task was slightly more difficult, since the sentences were longer (caused by an additional PP, see General Method section). Importantly, still 5 out of 15 HI children performed significantly below the hearing control group.

12 There seem to be some differences between German child language and for instance Hebrew child language in this respect, as discussed by Biran and Ruigendijk (2015).

13 Although simple SVO can be produced with raising only up to IP, and we would not be able to tell the difference by the phonological string.
