# Supplementary material for: A Deficit in Movement-Derived Sentences in German-Speaking Hearing-Impaired Children
Source: Front Psychol. 2017 Jun 13;8:689. doi: 10.3389/fpsyg.2017.00689 (PMC5468451; doi:10.3389/fpsyg.2017.00689)
Supplement: Supplementary file 1 [file Data_Sheet_1.docx]

#### Appendix A

Characteristics of the children who participated in the hearing impaired group – Experiments 1 and 2

| Age at implantation of first CI (2^nd^ CI) | Device | At special school? | Age at the beginning of intervention-hearing aid fitted | Age at diagnosis | Gender | Age at first test | Participant |
| --- | --- | --- | --- | --- | --- | --- | --- |
|  | 2HA | Yes | 5;0 | 5;0 | Male | 11;11 | 1 |
|  | 2HA | Yes | 0;6 | 0;6 | Male | 11;01 | 2 |
|  | 2HA | Yes | 3;0 | 3;0 | Female | 10;02 | 3 |
|  | 2HA | Yes | 3;0 | 2;0 | Female | 10;09 | 4 |
|  | 2HA | Yes | 5;0 | 4;0 | Male | 10;09 | 5 |
|  | CI(r) | Yes | 1;6 | 1;0 | Female | 9;11 | 6 |
|  | 2HA | Yes | 9;0 | 9;0 | Male | 9;11 | 7 |
|  | 2HA | Yes | 8;0 | 8;0 | Male | 13;0 | 8 |
| 8;0 | CI | Yes | 1;0 | 0;6 | Male | 10;03 | 9 |
|  | 2HA | Yes | 3;0 | 3;0 | Male | 10;01 | 10 |
| 5;0 (left 6;0) | 2CI | No | 3;0 (HA) | 3;0 | Male | 12;03 | 11 |
| 1;10 (left 5;0) | 2CI | No | 0;6 (HA) | 0;6 | Male | 9;05 | 12 |
|  | 2HA | No | 6;0 | 6;0 | Female | 11;09 | 13 |
|  | 2HA | No | 2;9 | 2;6 | Female | 10;07 | 14 |
|  | 2HA | No | 2;0 | 2;0 | Male | 10;11 | 15 |
| 6;0 | CI | Yes | 3;0 | 3;0 | Female | 10;5 | 16 |
|  | 2HA | Yes | 0;10 | 0;6 | Female | 9;4 | 17 |
|  | 2HA | Yes | 3;0 | 3;0 | Female | 10;8 | 18 |
|  | CI(r) | Yes | 2;6 | 2;6 | Female | 11;3 | 19 |

HA = hearing aid; CI = cochlear implant

####

Appendix B. Pairwise comparison of sentence type within each group (Bonferroni corrected) significant differences are marked bold.

|  | Hearing group | HI group |
| --- | --- | --- |
| svo vs passives | *p* = .71 | *p* = 1.0 |
| svo vs subject who | *p* = 1.0 | *p* = 1.0 |
| svo vs object who | *p* = .19 | ***p* = .01** |
| svo vs subject which | *p* = 1.0 | *p* = 1.0 |
| svo vs object which | *p* = .14 | ***p* = .01** |
| svo vs sr-rb | *p* = .40 | *p* = 1.0 |
| svo vs sr-ce | *p* = 1.0 | *p* = .25 |
| passives vs subject who | *p* = .38 | *p* = 1.0 |
| passives vs object who | ***p* = .02** | ***p* = .03** |
| passives vs subject which | *p* = .82 | *p* = 1.0 |
| passives vs object which | ***p* = .04** | ***p* = .03** |
| passives vs sr-rb | *p* = .13 | *p* = 1.0 |
| passives vs sr-ce | *p* = .52 | *p* = .36 |
| subject who vs object who | *p* = 1.0 | ***p* = .01** |
| subject who vs subject which | *p* = 1.0 | *p* = 1.0 |
| subject who vs object which | *p* = 1.0 | ***p* = .01** |
| subject who vs sr-rb | *p* = 1.0 | *p* = 1.0 |
| subject who vs sr-ce | *p* = 1.0 | *p* = .56 |
| object who vs subject which | *p* = .87 | ***p* = .05** |
| object who vs object which | *p* = 1.0 | *.* |
| object who vs sr-rb | *p* = 1.0 | ***p* = .03** |
| object who vs sr-ce | *p* = 1.0 | *p* = 1.0 |
| subject which vs object which | *p* = .91 | ***p* = .05** |
| subject which vs sr-rb | *p* = 1.0 | ***p* = .03** |
| subject which vs sr-ce | *p* = 1.0 | *p* = .59 |
| object which vs sr-rb | *p* = 1.0 | ***p* = .03** |
| object which vs sr-ce | *p* = 1.0 | *p* = 1.0 |
| sr-rb vs sr-ce | *p* = 1.0 | *p* = .72 |
